# Effects of Different Chemical Forms of Nitrogen on the Quick and Reversible Inhibition of Soybean Nodule Growth and Nitrogen Fixation Activity

**DOI:** 10.3389/fpls.2019.00131

**Published:** 2019-02-19

**Authors:** Natsumi Yamashita, Sayuri Tanabata, Norikuni Ohtake, Kuni Sueyoshi, Takashi Sato, Kyoko Higuchi, Akihiro Saito, Takuji Ohyama

**Affiliations:** ^1^Faculty of Agriculture, Niigata University, Niigata, Japan; ^2^College of Agriculture, Ibaraki University, Mito, Japan; ^3^Faculty of Bioresource Sciences, Akita Prefectural University, Akita, Japan; ^4^Faculty of Applied Biosciences, Tokyo University of Agriculture, Tokyo, Japan

**Keywords:** ammonium, glutamine, nodule growth, nitrate, nitrogen fixation activity, soybean, urea, ^15^N and ^13^C tracer experiment

## Abstract

It has been reported that supply of nitrate to culture solution rapidly and reversibly inhibits nodule growth and nitrogen fixation activity of soybean. In this study, the effects of ammonium, urea, or glutamine on nodule growth and nitrogen fixation activity are compared with that for nitrate. Soybean plants were cultivated with a nitrogen-free nutrient solution, then 1 mM-N of nitrate, ammonium, glutamine, or urea were supplied from 12 DAP until 17 DAP. Repression of nodule growth and nitrogen fixation activity at 17 DAP were observed by ammonium, urea, and glutamine like nitrate, although the inhibitory effects were milder than nitrate. The removal of nitrogen from the culture solutions after nitrogen treatments resulted in a recovery of the nodule growth. It was found that the glutamine treatment followed by N-free cultivation gave highest nitrogen fixation activity about two times of the control. Tracer experiments with ^15^N and ^13^C were performed to evaluate the translocation of N and C to the different tissues. Culture solutions containing a ^15^N-labeled nitrogen source were supplied from 21 DAP, and the whole shoots were exposed to ^13^CO_2_ for 60 min on 23 DAP, and plants were harvested on 24 DAP. The percentage distribution of ^15^N in nodules was highest for ammonium (1.4%) followed by glutamine (0.78%), urea (0.32%) and nitrate (0.25%). The percentage distribution of ^13^C in the nodules was highest for the control (11.5%) followed by urea (5.8%), glutamine (2.6%), ammonium (2.3%), and nitrate (2.3%). The inhibitory effects of nitrogen compounds appeared to be related to a decrease in photoassimilate partitioning in the nodules, rather than ^15^N transport into the nodules. The free amino acid concentrations after nitrogen treatments were increased in the nodules and leaves by nitrate, in the roots by ammonium, in the stems by urea, and the roots, stems, and leaves by glutamine treatment. The concentrations of asparagine, aspartate, and glutamine were increased after nitrogen treatments. By the long-term supply of nitrogen for 2-weeks, nitrate significantly increased the lateral roots and leaf growth. The long-term supply of urea and glutamine also promoted the lateral roots and leaf growth, but ammonium suppressed them.

## Introduction

Soybean plants can use both the nitrogen (N) fixed by root nodules and the N absorbed from the roots. However, it is well known that the development of root nodules and N_2_ fixation activity are repressed when the nodulated roots are exposed to high concentrations of combined form of nitrogen, especially nitrate, a major chemical form of inorganic nitrogen in upland fields (Gibson and Harper, 1985; Imsande, 1986; Streeter, 1988; Ohyama et al., 2012). It was suggested that multiple effects of nitrate inhibition occur, such as the decreases in nodule number, nodule mass, and N_2_ fixation activity, as well as the acceleration of nodule senescence or disintegration, so nitrate inhibition cannot be explained in a simple way (Harper, 1987; Streeter, 1988). In addition, the effects of nitrate on nodule growth are influenced by nitrate concentration, placement in the medium, and treatment period (Yashima et al., 2003, 2005) as well as legume species (Harper and Gibson, 1984; Davidson and Robson, 1986).

Many hypotheses have been proposed regarding the causes of nitrate inhibition of nodulation and nitrogen fixation, such as carbohydrate-deprivation in nodules, feedback inhibition by a product of nitrate metabolism such as Asn or ureides (allantoic acid and allantoin) (Serraj et al., 1999, Vadez et al., 2000), and decreased oxygen diffusion into the nodules which restricts the respiration of bacteroides (Schuller et al., 1988; Vessey et al., 1988; Gogorcena et al., 1997; Gordon et al., 2002).

Direct (local) supply of nitrate to the nodulated part of the roots and indirect (systemic) supply from the distal part of the roots have elicited different responses of nodule growth and nitrogen fixation activity (Yashima et al., 2003, 2005). Further, direct effect of 5 mM nitrate on growth and nitrogen fixation activity of the nodules was quick and reversible in hydroponically cultured soybean seedlings (Fujikake et al., 2002, 2003). The diameter of the individual nodule was measured by a slide caliper under the controlled environmental conditions from 10 to 24 DAP. The diameter of a root nodule attached to the primary root increased from 1 to 6 mm for 2 weeks in N-free culture conditions. The increase in diameter of the nodule was almost completely stopped after 1 day of supplying 5 mM nitrate. However, nodule growth quickly returned to the normal growth rate following the withdrawal of nitrate from the solution.

The effects of nitrate on soybean nodule growth have been monitored at 1-h intervals, and the growth of nodules was measured with newly developed computer software (Saito et al., 2014; Tanabata et al., 2014). Nodule growth began to be suppressed quickly at several hours after the addition of 5 mM of nitrate compared with control plants. Similar repressive effects were observed on the growth rate of the primary roots. Conversely, the growth rate of soybean lateral roots was promoted by the addition of 5 mM of nitrate (Saito et al., 2014).

Plant leaves have been exposed to ^11^C or ^14^C-labeled carbon dioxide to investigate the effect of 5 mM nitrate on photosynthate translocation and distribution to nodules and roots (Fujikake et al., 2003). The supply of 5 mM nitrate stimulated the translocation rate and the distribution of labeled-C in the nitrate-fed part of the roots. However, the ^14^C partitioning to nodules markedly decreased. These results indicated that a decrease in photoassimilate supply to the nodules might be involved primarily in the quick and reversible nitrate inhibition of soybean nodule growth. A question remains, however, as to whether a quick and reversible effect of nitrate inhibition on nodule growth and nitrogen fixation is specific for nitrate, or might a similar effect occur for application of the other nitrogen sources such as ammonium sulfate, urea, or Gln. In addition, the inhibitory effect of the nitrogen compound might be caused directly by the accumulation of nitrogen compounds in nodules or via changes in photoassimilate partitioning in nodules as in the case of nitrate inhibition.

Amino acids are the key metabolites in nitrogen (N) metabolism of higher plants (Ohyama et al., 2017). First, the inorganic N such as ammonium absorbed in the roots or produced from nitrate reduction, nitrogen fixation in root nodules and photorespiration in leaves, are initially assimilated into Gln and glutamate (Glu) by GS/GOGAT pathway. Second, amino acids are the essential components of proteins. Third, amino acids are used for a long distance transport of nitrogen among organs (roots, nodules, stems, leaves, pod, seeds, apical buds) through xylem or phloem. Forth, non-protein amino acids may play a role in protecting plants from feeding damages by animals, insects or infection by fungi.

The nitrogen assimilation in soybean nodules, the time course experiment with ^15^N_2_ feedings in the nodulated intact soybean plants with specific inhibitors revealed that the ammonia produced by nitrogen fixation in bacteroid is rapidly released to the cytosol of the infected cells and is initially assimilated into amide group of Gln by the enzyme GS, then the Gln and 2-OG produce two moles of Glu by the enzyme glutamate synthase (GOGAT) (Ohyama and Kumazawa, 1978, 1980a). Some part of Gln is used for purine base synthesis and uric acid is transported from the infected cells to the adjacent uninfected cells in the central symbiotic region of nodule. Uric acid is catabolized into allantoin and allantoic acid in the uninfected cells, then transported to the shoot through xylem vessels in the roots and stems. A small portion of fixed N was assimilated into Ala and Glu in the bacteroides, but it was not metabolized by GS/GOGAT pathway (Ohyama and Kumazawa, 1980b). It is established that the ureides are the principal N transport compounds from soybean nodules, but Asn is also transported from nodules. Huber and Streeter (1984) reported that Gln-dependent AS catalyses the amidation of aspartate to Asn in the cytosol fraction of infected zone of soybean nodules. Minamisawa et al. (1986) estimated the Asn and ureide pools in soybean nodules after ^15^N_2_ exposure to the nodulated roots for 5.5 h, and revealed that fixed N in the transport form of Asn-N was about 1/5 of ureide-N.

In the present research, nodulated soybean seedlings were supplied with sodium nitrate, ammonium sulfate, urea, or Gln for 5 days from 12 to 17 DAP, and the effects of nitrogen supply on nodule growth and nitrogen fixation activity as measured by ARA were determined on 17 DAP. Control plants were grown with an N-free culture solution throughout the experimental period. In a further study, plants were grown continuously in an N-free solution for 7 days from 17 to 24 DAP after 5 days of nitrogen treatments. In this series of experiments, nodule growth was measured every day, and the effect of the different nitrogen compounds on nodule volume was evaluated.

In a second experiment, a ^13^C and ^15^N double tracer experiment was conducted. Culture solutions containing 1 mM-N of ^15^N-labeled Na^15^NO_3_, (^15^NH_4_)_2_SO_4_, ^15^N-urea, or ^15^N-Gln were supplied for 3 days from 21 to 24 DAP. The whole shoot of the ^15^N-supplied plant was enclosed in a plastic bag on 23 DAP, and the leaves in the plastic bag were exposed to ^13^CO_2_. At 26 h after ^13^CO_2_ exposure, plants were harvested on 24 DAP. Control plants were grown in a N-free culture solution throughout the experiment period. The plants were freeze-dried and then separated into nodules, roots, stems, and leaves. The tissue samples corresponding to each part were ground into a fine powder and the ^15^N and ^13^C enrichment in these parts were determined. The free amino acids in the nodules, roots, stems, and leaves of the plants treated with ^15^N and ^13^C were extracted with 80% ethanol before analysis using a Waters Aquity UPLC system.

In a final experiment, a long-term effect on the root architectures and whole plant growth with supplying N compounds for 2 weeks were observed The 20-day-old soybean plants were treated with the culture solutions containing 1 mM NaNO_3_, 0.5 mM (NH_4_)_2_SO_4_, 0.5 mM urea or 0.5 mM Gln for 2 weeks. Three lateral roots were marked with the color strings, and the main root length, marked lateral roots length and nodule diameter were measured at 1 and 2 weeks during treatments. After treatments, the plants were separated into roots and shoots, and the root length and leaf area were analyzed by the plant image analyzer. Plants were dried in a ventilation oven and dry weight of each tissue was measured.

## Materials and Methods

### Plant Material and Growth Conditions

Soybean [*Glycine max* (L.) Merr. cv. Williams] seeds were inoculated with a suspension of *Bradyrhizobium diazoefficiens* (strain USDA 110) and sown in vermiculite. After 5 DAP, each seedling was transplanted to a glass bottle with 800 mL of N-free nutrient solution (Fujikake et al., 2002). The culture solution was continuously aerated by an air-pump and changed three times a week. Plants were cultivated in a climate chamber (28/18°C day/night temperature, 55% relative humidity, 228 μmol m^-2^ s^-1^ PPFD, 16/8 h day/night photoperiod).

### The Effects of the Application of Nitrogen Compounds on Nodule Growth

Culture solutions containing 1 mM nitrogen source [1 mM NaNO_3_, 0.5 mM (NH_4_)_2_SO_4_, 0.5 mM urea or 0.5 mM Gln] were supplied for 5 days from 12 to 17 DAP (Experiment 1). The solution was renewed every day. Control plants were grown with an N-free culture solution throughout the experimental period. The ARA was measured on 17 DAP, then the dry weight of each tissue was determined.

In the second experiment (Experiment 2), the plants were cultivated with the various nitrogen compounds (same as in the Experiment 1) and then were continually grown with a N-free solution from 17 to 24 DAP. Control plants were grown with an N-free culture solution from 12 to 24 DAP. The diameter (d) of the horizontal axis of five selected nodules attached to the main root per single plant was measured by a slide caliper every day, then the effects of nitrogen compounds on nodule growth were evaluated from the changes in nodule volume. The volume of each nodule was calculated by the formula for the volume of a sphere 4πr^3^/3, where r is the radius of a nodule (r = d/2). Then the ARA was measured on 24 DAP, and the dry weight of each tissue was determined. The experiment was replicated four individual plants. Statistical analysis was performed using Tukey’s test.

### Acetylene Reduction Activity

The nodulated root of each plant in Experiments 1 and 2 was separated from the shoot and immediately incubated with 10% (V/V) acetylene in a 600 ml glass bottle at 25°C for 20 min, then the ethylene produced by nitrogenase was monitored by a gas chromatography equipped with FID (Gas Chromatograph 163, Hitachi, Tokyo, Japan) fitted with a Porapack N column (GL Science, Tokyo, Japan). We could not detect the wound-induced ethylene production without acetylene in this system.

### ^13^C and ^15^N Double Tracer Experiment

Soybean plants were cultivated in a solution culture with an N-free solution until 21 DAP, and different forms of nitrogen compounds were supplied for 3 days when the inhibitory effect on nodule growth became apparent in Experiment 2 (Experiment 3). Culture solution containing ^15^N-labeled 1 mM nitrogen sources (1 mM Na^15^NO_3_; 6.82 atom%, 0.5 mM (^15^NH_4_)_2_SO_4_; 7.07 atom%, 0.5 mM ^15^N-urea; 5.08 atom%, or 0.5 mM amide, amino ^15^N-Gln; 8.67 atom%), were supplied from 21 to 24 DAP. The solutions were renewed every day. On 23 DAP, the whole shoot of a plant was enclosed in a plastic bag (Gas volume about 2 L, 270 × 200 mm). Two mL of ^13^CO_2_ (99.0 atom%) was injected into each plastic bag and the CO_2_ concentration in the bag was monitored by an infrared CO_2_ analyzer (Testo535, Testo Co. Ltd., Yokohama, Japan) (Supplementary Figure S1). At 30 min after the first injection, the ^13^CO_2_ was almost depleted, thus a further 2 mL of ^13^CO_2_ was injected. Plants were exposed to the ^13^CO_2_ for 60 min in total, then the plastic bag was removed. At 26 h after ^13^CO_2_ exposure, plants were harvested on 24 DAP. Control plants were grown with an N-free culture solution throughout the experimental period. The plants were immediately frozen with liquid nitrogen and dried in a freeze-drier. The plants were separated into leaves, stems, roots, and nodules. The samples of each part were ground into a fine powder by a vibratory mill. The ^15^N and ^13^C enrichment in tissue parts were determined using an Elemental analyzer (EA1110, Thermo Electron) -IRMS coupled system (DELTA Plus Advantage, Thermo Electron, Boston, MA, United States).

### Analysis of Free Amino Acids

The free amino acids in the nodules, roots, stems and leaves of the plants treated with ^15^N and ^13^C (Experiment 3) were extracted with 80% (V/V) ethanol, then derivatized with AQC (6-aminoquinolyl-*N*-hydroxysuccinimidyl-carbamate) reagent, and analyzed by a Waters Aquity UPLC system equipped with a Waters AccQ Tag Ultra column (Waters, Milford, MA, United States) (Nagumo et al., 2009).

### Long-Term Effects of Various N Compounds on Root Architecture and Plant Growth

Twenty-day-old soybean plants were treated with the culture solutions containing 1 mM NaNO_3_, 0.5 mM (NH_4_)_2_SO_4_, 0.5 mM urea or 0.5 mM Gln for 2 weeks (Experiment 4). Culture solutions were renewed every 2 days. The root structure and shoot phenotypes were monitored after 2-weeks treatments. Three lateral roots were marked with the color strings tied above a nodule, and the main root length, marked lateral root length and diameter of nodules attached to the lateral roots were measured at 1 and 2 weeks during treatments. The chlorophyll concentration (SPAD values) after 2-week treatments were determined for second, third, and fourth leaves by Chlorophyll meter SPAD-502 (Konica Minolta Sensing, Inc., Japan). The plants were separated into roots and shoots, and the root length and leaf area were analyzed by the plant image analyzer (WinRHIZO, STD 4800, Regent Instruments Inc., Canada). Plant samples were dried in a ventilation oven and dry weight of each tissue was measured.

## Results

### Effects of Various Nitrogen Compounds on Plant Growth and Acetylene Reduction Activity

**FIGURE 1:**
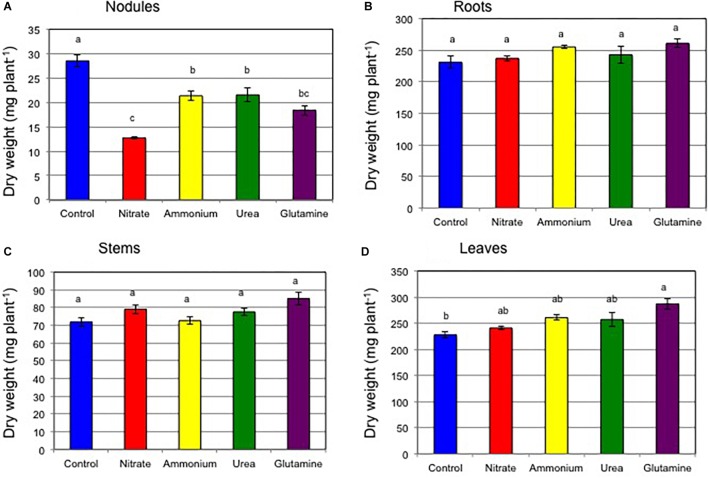
Comparison of the dry weight of each plant tissue at 17 DAP supplied with control (N-free), nitrate, ammonium, urea, or glutamine from 12 to 17 DAP (Experiment 1). **(A)** Nodules. **(B)** Roots. **(C)** Stems. **(D)** Leaves. Averages and standard errors are shown (*n* = 4). Different letters above the column indicate significant differences at <0.05 by Tukey’s test.

Figure 1 shows the dry weight for each plant tissue on 17 DAP after various nitrogen treatments for 5 days (Experiment 1). The total plant dry weight was 559 mg (control), 570 mg (nitrate), 610 mg (ammonium), 599 mg (urea), and 651 mg (Gln). The dry weights of roots (Figure 1B), stems (Figure 1C), and leaves (Figure 1D) of plants with N compounds were similar or slightly higher than those of the control plants. In the case of the dry weights of nodules, the values were repressed by the supply of 1 mM-N for 5 days compared with control plants (Figure 1A). The dry weight values for nodules were 28.5 mg (control), 12.7 mg (nitrate), 21.4 mg (ammonium), 21.6 mg (urea), and 18.4 mg (Gln). Based on Tukey’s test, the nodule dry weight for the control plant was significantly different (*P* < 0.05) compared to all the nitrogen treated plants. The addition of the nitrogen compounds resulted in reductions of nodule dry weight of 45% (nitrate), 75% (ammonium), 76% (urea), and 65%, (Gln) relative to the control plants, respectively. The repressive effect of nitrogen compounds was the strongest for nitrate, while weaker repression occurred for additions of ammonium, urea, and Gln. The numbers of nodules per plant, 48.5 (control), 43.8 (nitrate), 45.8 (ammonium), 43.8 (urea), and 39.5 (Gln), were not significantly different among the four different nitrogen treatments (Supplementary Figure S2).

**FIGURE 2:**
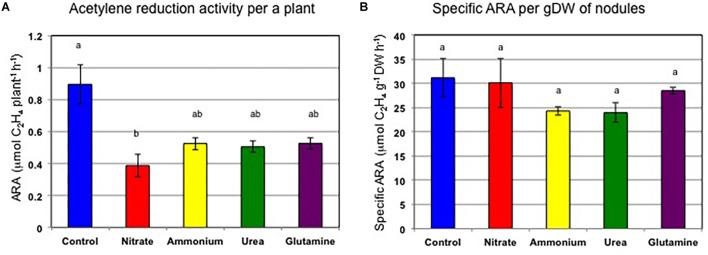
Acetylene reduction activity per single plant, and specific acetylene reduction activity per nodule g dry weight on 17 DAP supplied with control (N-free), nitrate, ammonium, urea, or glutamine from 12 to 17 DAP for 5 days (Experiment 1). **(A)** ARA per single plant. **(B)** Specific ARA per g dry weight of nodules. Averages and standard errors are shown (*n* = 4). Different letters above the column indicate significant differences at <0.05 by Tukey’s test.

Figure 2 shows the ARA per a single plant and the specific ARA per nodule dry weight. The average ARA per plant was 0.90 μmol ethylene formed per h (control), 0.39 μmol (nitrate), 0.53 μmol (ammonium), 0.50 μmol (urea), and 0.53 μmol (Gln) (Figure 2A). The addition of nitrogen compounds resulted in a reduction in the ARA of 43% (nitrate), 59% (ammonium), 56% (urea), and 59% (Gln) relative to control plants, respectively. Based on Tukey’s test, the ARA for the control plant was significantly different compared to that for nitrate treatment. The specific ARAs per g dry weight of nodules were 31.1 μmol ethylene formed per h per g dry weight (control), 30.1 μmol (nitrate), 24.3 μmol (ammonium), 23.9 μmol (urea), and 28.5 μmol (Gln) (Figure 2B). The addition of nitrogen compounds resulted in a reduction of the specific ARA to 97% (nitrate), 78% (ammonium), 77% (urea), and 92%, (Gln) relative to the control plants, respectively. The specific ARAs were not significantly different for the different nitrogen treatments according to Tukey’s test. These findings suggested that the repression of the total ARA by the different N treatments was mainly due to a decrease in nodule weight rather than a specific ARA effect.

### The Effects of Application of Various Chemical Forms of Nitrogen From 12 to 17 DAP Followed by Cultivation With an N-Free Solution

**FIGURE 3:**
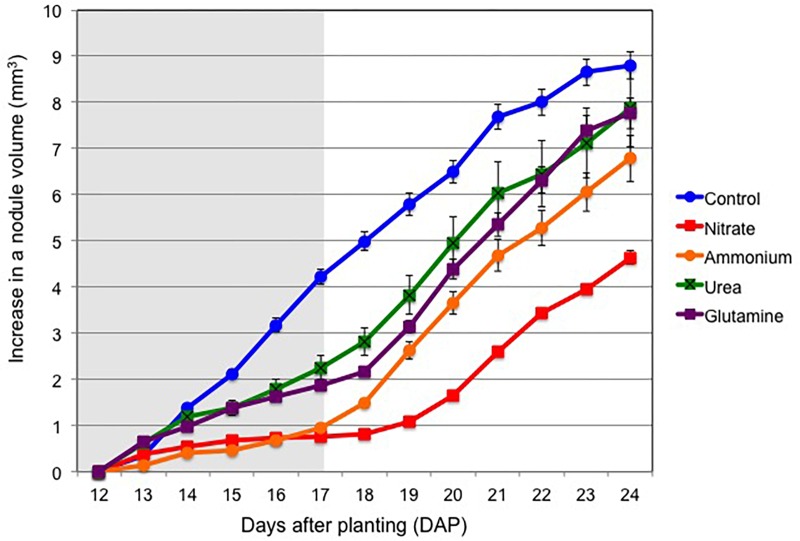
Changes in nodule volume from 12 to 24 DAP for treatments with control (N-free), nitrate, ammonium, urea, or glutamine from 12 to 17 DAP, thereafter cultivated with a N-free culture solution (Experiment 2). Shaded background indicates N treatment period, and white background indicates cultivation with N-free medium. Average and standard error are shown (*n* = 5).

In a second experiment, plants were cultivated with various forms of nitrogen compounds from 12 to 17 DAP and then cultivation was continued with an N-free solution from 17 to 24 DAP in all treatments. Figure 3 shows the changes in nodule volume from 12 to 24 DAP. The control soybean nodules grew steadily from 12 to 24 DAP in an N-free nutrient solution. When 1 mM nitrate was supplied at 12 DAP, the nodule growth was quickly repressed from the next day after nitrate addition, and stopped completely after the second day on 14 DAP. The addition of ammonium also quickly repressed the nodule growth from the first day and the increase in the nodule volume was less than half of that in the control on the third day on 15 DAP. The addition of urea and Gln resulted in similar growth patterns. The nodule growth was slightly repressed after 14 DAP, but the repression was weaker compared with that for the nitrate and ammonium treatments. After replacing the N-based culture solutions with the N-free solution on 17 DAP, the nodule growths for all treatments showed quick recoveries, although there was a longer time-lag of about 2 days after the addition of nitrate.

**FIGURE 4:**
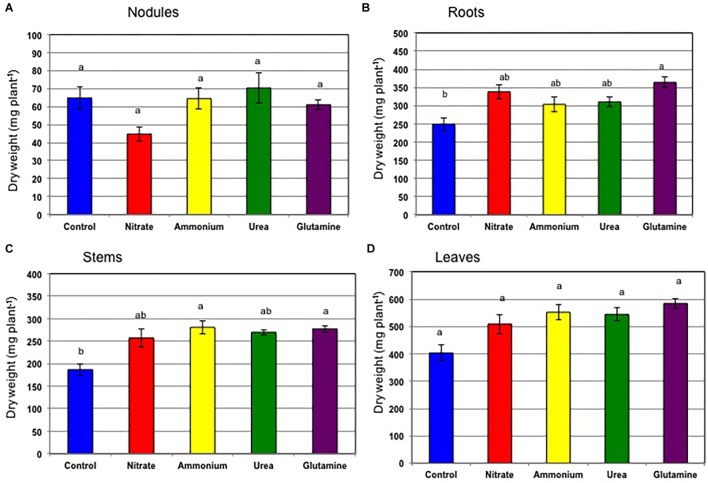
Comparison of the dry weight of each plant tissue on 24 DAP for treatment with control (N-free), nitrate, ammonium, urea, or glutamine from 12 to 17 DAP, thereafter cultivated with a N-free culture solution (Experiment 2). **(A)** Nodules. **(B)** Roots. **(C)** Stems. **(D)** Leaves. Averages and standard errors are shown (*n* = 4). Different letters above the column indicate significant differences at <0.05 by Tukey’s test.

Figure 4 shows the dry weight of each plant tissue on 24 DAP after various nitrogen treatments for 5 days from 12 to 17 DAP and with continued growth in the N-free medium from 17 to 24 DAP (Experiment 2). The total plant dry weights were 904 mg (control), 1,150 mg (nitrate), 1,200 mg (ammonium), 1,195 mg (urea), and 1,290 mg (Gln). The dry weights of roots, stems, and leaves of plants receiving the various N treatments were slightly higher than those of the control plants. Compared with nodule dry weight just after N treatment on 17 DAP (Figure 1A), the dry weights of nodules of the plants with N were recovered compared with control plants on 24 DAP after continued growth in the N-free medium for 7 days (Figure 4A). The dry weights of the nodules were 64.8 mg (control), 44.8 mg (nitrate), 64.5 mg (ammonium), 70.3 mg (urea), and 68.8 mg (Gln); the nodule dry weights were not significantly different among all treatments according to Tukey’s test. The addition of nitrogen compounds from 12 to 17 DAP tended to increase the dry weights of roots (Figure 4B), stems (Figure 4C), and leaves (Figure 4D) at 24 DAP.

**FIGURE 5:**
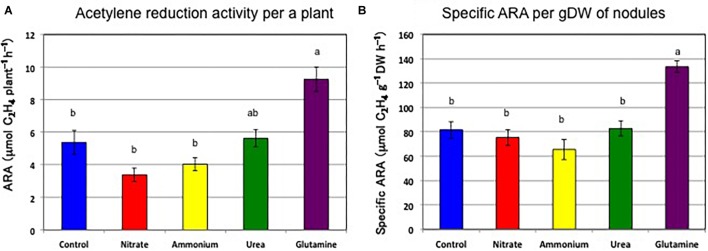
Acetylene reduction activity per single plant and specific acetylene reduction activity per nodule g dry weight on 24 DAP for treatments with control (N-free), nitrate, ammonium, urea, or glutamine for 5 days from 12 to 17 DAP with continuation of growth from 17 to 24 DAP with N-free medium (Experiment 2). **(A)** ARA per single plant. **(B)** Specific ARA per g dry weight of nodules. Averages and standard errors are shown (*n* = 4). Different letters above the column indicate significant differences at <0.05 by Tukey’s test.

Figure 5 shows the ARA per single plant and the specific ARA per g nodule dry weight on 24 DAP. The average ARAs per single plant were 5.4 μmol (control), 3.4 μmol (nitrate), 4.0 μmol (ammonium), 5.6 μmol (urea), and 9.2 μmol (Gln) ethylene formed per h per g nodule dry weight (Figure 5A). The ARA levels recovered to levels near that of the control for cultivation with the N-free solution after the addition of the nitrogen compounds. Unexpectedly, the addition of Gln from 12 to 17 DAP increased significantly the ARA (172%) on 24 DAP compared to the treatment for the control. The specific ARA were 81.6 μmol (control), 75.4 μmol (nitrate), 65.5 μmol (ammonium), 82.8 μmol (urea), and 133.5 μmol (Gln) ethylene formed per h per g dry weight of nodules (Figure 5B), and the specific ARAs were significantly different for treatment by Gln compared to the other nitrogen compounds according to Tukey’s test.

### ^15^N and ^13^C Double Tracer Experiment

**FIGURE 6:**
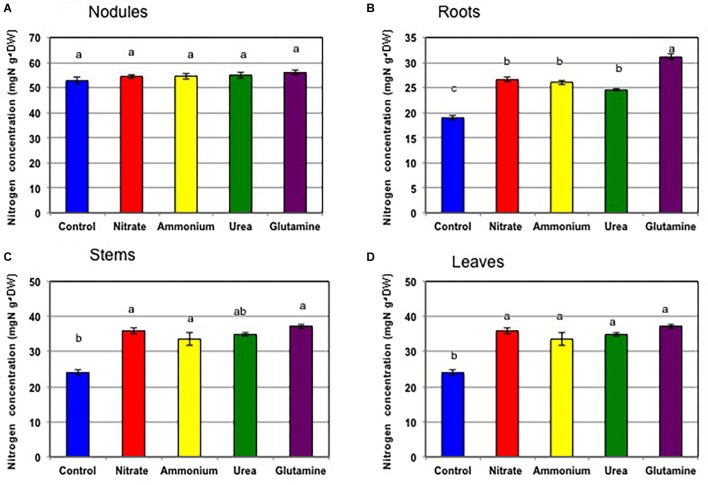
Nitrogen concentration in tissues of soybean plants treated with control (N-free), nitrate, ammonium, urea, or glutamine for 3 days from 21 to 24 DAP (Experiment 2). **(A)** Nodules. **(B)** Roots. **(C)** Stems. **(D)** Leaves. Averages and standard errors are shown (*n* = 4). Different letters above the column indicate significant differences at <0.05 by Tukey’s test.

Supplementary Table S1 shows the dry weight of each tissue of soybean plants for ^15^N and ^13^C double tracer experiment. The nodule dry weights of the plants with various N compounds were relatively low compared with that of the control plants although statistically not significant according to Tukey’s test. Figure 6 shows the nitrogen concentrations in each tissue of the soybean plant at 24 DAP after cultivation with various N compounds for 3 days from 21 to 24 DAP. The nitrogen concentrations in nodules were similar about 53–56 mgN g^-1^ dry weight for the various treatments including that for the control plants (Figure 6A). In contrast, the nitrogen concentrations of roots (Figure 6B), stems (Figure 6C), and leaves (Figure 6D) were significantly higher in the plants supplied with N compounds. The nitrogen levels in the roots treated with Gln had the highest concentrations at 31 mgN g^-1^ dry weight, the levels being significantly higher than that for the other treatments.

**FIGURE 7:**
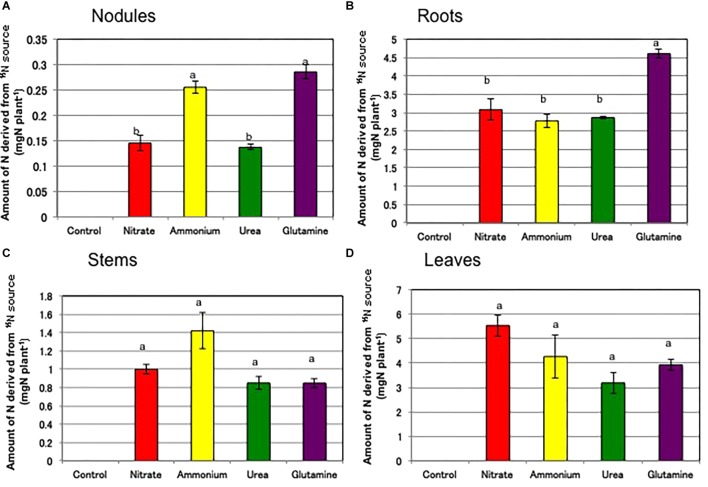
Amounts of N derived from ^15^N-labeled source in each tissue of soybean plants on 24 DAP supplied for 3 days from 21 to 24 DAP (Experiment 3). **(A)** Nodules. **(B)** Roots. **(C)** Stems. **(D)** Leaves. Averages and standard errors are shown (*n* = 4). Different letters above the column indicate significant differences among each treatment at <0.05 by Tukey’s test.

Figure 7 shows the amounts of N in each tissue derived from the ^15^N-labeled sources supplied for 3 days from 21 to 24 DAP. The culture solutions containing 11.3 mg of labeled N, were changed every day, so a total of 33.6 mg of ^15^N-labeled N was supplied during the period of the labeling experiment. The total amounts of N derived from the ^15^N-labeled source were 0 mg (control), 9.75 mg (nitrate), 8.71 mg (ammonium), 7.04 mg (urea), and 9.67 mg (Gln), respectively. These results indicated that urea and Gln as well as nitrate and ammonium were absorbed efficiently from the roots, although urea absorption tended to be lower than that of the other compounds; this finding, however, was not statistically significant. The ^15^N-labeled N was not depleted from the culture solution, because the daily supply of labeled N was 11.3 mg, which was higher than the total amount of ^15^N absorbed by a plant in the 3 days. The amounts of N derived from ^15^N in the nodules (Figure 7A) were 0 mg (control), 0.14 mg (nitrate), 0.26 mg (ammonium), 0.14 mg (urea), and 0.29 mg (Gln). The amounts of N from Gln and ammonium were significantly higher than those from nitrate and urea. The amounts of N derived from ^15^N in the roots (Figure 7B) were 0 mg (control), 3.08 mg (nitrate), 2.78 mg (ammonium), 2.87 mg (urea), and 4.61 mg (Gln). The amount of N from Gln was significantly higher than that from nitrate, ammonium, or urea. The amount of N from ^15^N in the stems tended to be high in the stem treated with ammonium (Figure 7C), and that was high in the leaves treated with nitrate (Figure 7D).

The percentage distribution of ^15^N (Supplementary Table S2) in the shoots (stems + leaves) was highest for nitrate (67%), and ammonium (65%) treatments followed by urea (57%) and then Gln (49%). The percentage distribution of ^15^N in the leaves was highest for nitrate treatment (57%), followed by ammonium treatment (49%), urea (45%) and then Gln (41%). In contrast, the percentage distribution of ^15^N in the stems was highest for ammonium treatment (16%), followed by urea (12%), nitrate (10%) and Gln (9%). The percentage distribution of ^15^N in the roots was highest for Gln treatment (48%), followed by urea (41%), and ammonium (32%) and then nitrate (32%). The percentage distribution of ^15^N in the nodules was higher for Gln (3.0%) and ammonium (2.9%) relative to that for nitrate (1.5%) and urea (1.9%).

**FIGURE 8:**
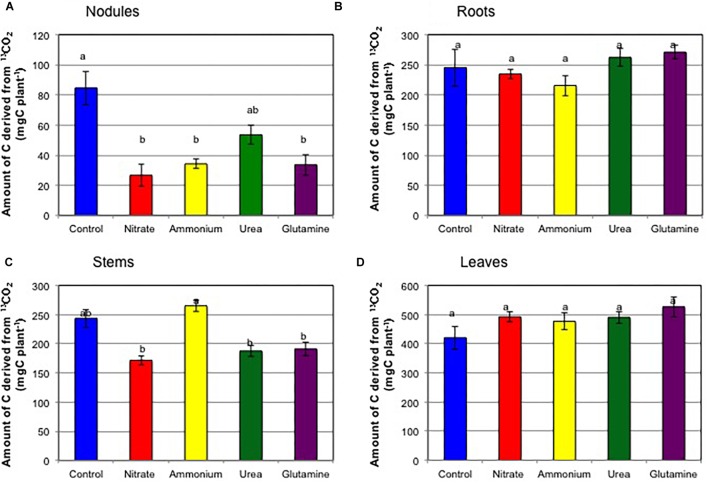
Amounts of C derived from ^13^C-labeled CO_2_ in each tissue of soybean plants on 24 DAP supplied for 1 h at 23 DAP (Experiment 3). **(A)** Nodules. **(B)** Roots. **(C)** Stems. **(D)** Leaves. Averages and standard errors are shown (*n* = 4). Different letters above the column indicate significant differences among each treatment at <0.05 by Tukey’s test.

The total contents of C derived from ^13^C-labeled CO_2_ were 993 μg (control), 925 μg (nitrate), 991 μg (ammonium), 993 μg (urea), and 1021 μg (Gln). About 2000 μg of ^13^C was exposed to each plant in the form of ^13^CO_2_, so about one half of the supplied ^13^C remained in the plant at 1 day after ^13^CO_2_ exposure. The other half may not be fixed during incubation period, and be lost by respiration of the plants during 26 h after ^13^CO_2_ exposure. Figure 8 shows the amounts of C derived from ^13^C-labeled CO_2_ in the tissues of the soybean plants supplied for 1 h at 23 DAP. The amounts of C from ^13^C in the nodules (Figure 8A) were 85 μg (control), 27 μg (nitrate), 34 μg (ammonium), 54 μg (urea), and 34 μg (Gln). The addition of nitrogen compounds resulted in the reductions in the amounts of C derived from ^13^C, the relative reductions to the control (100%) being 32% (nitrate), 40% (ammonium), 64% (urea), and 40% (Gln), respectively. The amounts of C from ^13^C in the nodules were significantly different for the control and the other treatments except for urea. The amounts of C from ^13^C were not significantly different in the roots (Figure 8B) and the leaves (Figure 8D) for the different nitrogen treatments. The amount of C derived from ^13^C was significantly high in the stems treated with ammonium compared with nitrate, urea, and Gln (Figure 8C).

Supplementary Table S3 shows the percentage distribution of ^13^C for the various N treatments. The percentage distribution of ^13^C in the nodules was highest for the control treatment (11.5%) followed by urea (5.8%), Gln (2.6%), ammonium (2.3%), and nitrate (2.3%). The amounts of ^13^C in the nodules (Figure 8A) and the percentage distributions of ^13^C in the nodules were in accordance with the decrease in nodule growth (Figure 1, 3) and the ARAs (Figure 2). The percentage distribution of ^13^C in the roots was the lowest for the ammonium treatment (19.7%), but this treatment also resulted in the highest percentage distribution of ^13^C in the stem (30.6%). The percentage distribution of ^13^C in the leaves was highest for nitrate treatment (56.7%) followed by urea (50.1%) and Gln (50.1%), ammonium (47.4%), the lowest value being in the in control (35.4%).

### Free Amino Acid Concentration in Soybean Tissue

**FIGURE 9:**
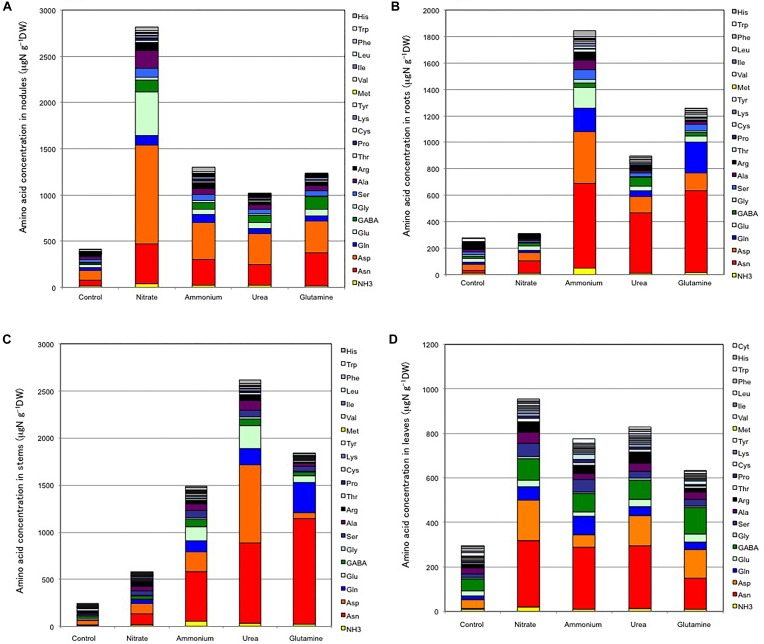
Free amino acid concentrations in each tissue of soybean plants on 24 DAP supplied with various N compounds from 21 to 24 DAP (Experiment 3). **(A)** Nodules. **(B)** Roots. **(C)** Stems. **(D)** Leaves. Averages are shown (*n* = 4).

Figure 9 shows the free amino acid concentrations in the nodules (Figure 9A), roots (Figure 9B), stems (Figure 9C), and leaves (Figure 9D) at 24 DAP after 3 days of nitrogen supply. For the nodules (Figure 9A), the total amino acid N concentration was lowest in the control (410 μgN g^-1^ dry weight), and highest for nitrate supplementation (2,820 μgN g^-1^ dry weight), with intermediate values being obtained for ammonium (1,300 μgN g^-1^ dry weight), urea (1,020 μgN g^-1^ dry weight) and Gln (1,240 μgN g^-1^ dry weight) treatments. In the control nodules Asp, Asn, Ala, Ser, Glu, and Gln were the major free amino acids. In the nodules obtained for nitrate supplementation, there were about a sevenfold increase in total free amino acid concentration relative to that for the control nodules, and Asp, Glu, Asn, Ala, and GABA were the major amino acids. In the nodules supplemented with ammonium, urea, and Gln the increase was about three times that for the control nodules, and Asp, Asn, Glu, Gln, GABA, and Ser were the major amino acids for these treatments, the relative composition of the amino acid N being not markedly different among the treatments. The Gln concentration in the nodules treated with Gln did not increase excessively.

The total free amino acid N concentrations in the roots (Figure 9B) were lowest for the control roots (273 μgN g^-1^ dry weight), and highest in the roots treated with ammonium (1,840 μgN g^-1^ dry weight), followed by Gln (1,260 μgN g^-1^ dry weight), urea (890 μgN g^-1^ dry weight), and nitrate (310 μgN g^-1^ dry weight). In the control roots, Asp, Glu, Asn, Ser, Ala, and Gln were major free amino acids. The Supply of nitrogen compounds increased Asn and Asp irrespective of the chemical form of nitrogen supplied. The supply of Gln and ammonium also increased the Gln concentration, although the level was much less than that of Asn.

The total free amino acid N concentrations in the stems (Figure 9C) were the lowest for the control stems (244 μgN g^-1^ dry weight), and highest in the stems treated with urea (2,610 μgN g^-1^ dry weight), followed by Gln (1,840 μgN g^-1^ dry weight), ammonium (1,490 μgN g^-1^ dry weight) and nitrate (580 μgN g^-1^ dry weight). In the control stems, Asp, GABA, and Cys were the major free amino acids. In general, irrespective of the chemical form of nitrogen, the supply of nitrogen compounds increased the concentration of Asn and Asp, the same as that found for the roots. The supply of Gln increased the Gln concentration in the stems.

The total free amino acid N concentrations in the leaves (Figure 9D) were lowest in the control leaves (293 μgN g^-1^ dry weight), and highest in the leaves treated with nitrate (950 μgN g^-1^ dry weight), followed by urea (820 μgN g^-1^ dry weight), ammonium (750 μgN g^-1^ dry weight), and Gln (630 μgN g^-1^ dry weight). In the control leaves, GABA, Asp, Ala, Arg, and Gln were the major free amino acids. The supply of nitrogen compounds increased Asn, Asp, GABA, Gln and Ser, irrespective of the chemical form of the supplied nitrogen. The supply of Gln did not increase the Gln concentration in the leaves.

The concentrations (Supplementary Table S4) and the relative concentrations (Supplementary Table S5) of free amino acids in each tissue for the various nitrogen treatments relative to those in the control plants were calculated. The relative concentrations of free ammonium were relatively high in the stems treated with ammonium (×8.6), urea (×5.5) and Gln (×4.2), and in the roots treated with ammonium (×4.9). Among the free amino acids, the relative concentrations of Asn were very high in roots (×4–25), stems (×9–80), leaves (×21–45), and nodules (×3.4–6.6). The relative concentrations of Asp were high especially in the nodules treated with nitrate (×10), in the roots treated with ammonium (×9.5), and in the stems treated with urea (×20). The relative concentrations of Gln were high especially in the stems (×24) and roots (×14) treated with Gln. The relative concentrations of Glu were high in the nodules treated with nitrate (×13), in the stems treated with urea (×25), ammonium (×15) and Gln (×8). The relative concentrations of GABA were high in the nodules for treatments with all nitrogen compounds and values were less the roots, stems, and leaves. The relative concentrations of Cys were markedly enhanced in the nodules and leaves but repressed in the stems. The relative concentrations of His were increased in the nodules, roots, and stems but decreased in the leaves. The relative concentrations of Trp in most tissue parts decreased as a result of the nitrogen treatments. The same was true in the case of Leu, Met, and Tyr in the leaves, Cys in the stems, and Met in the roots.

### Long-Term Effects of Various N Compounds From 20 to 34 DAP on Root Architecture and Plant Growth

**FIGURE 10:**
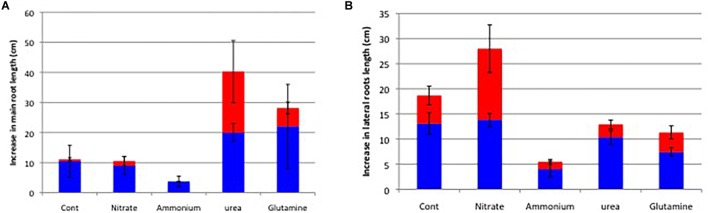
Increase in main roots **(A)** and the selected lateral roots **(B)** after the first week from 20 to 27 DAP (Blue bar), and the second week from 27 to 34 DAP (Red bar) of the treatments with various forms of N compounds (Experiment 4).

Figure 10 shows the increase in the main root length (Figure 10A) and marked three lateral root length (Figure 10B) on 34 DAP after various nitrogen treatments for 14 days from 20 to 34 DAP (Experiment 3). The main root length increased about 10 cm for the first week period of control and nitrate treatments from 20 to 27 DAP, but those for the second week period from 27 to 34 DAP was almost completely stopped. The application of ammonium most severely inhibited the main root growth for the first and second weeks. Contrary, the application of urea and Gln continued to increase the main root length for the first and second period of treatments. The average length of marked three lateral roots was about 13 cm and 6 cm during the first and second weeks in control treatment. Those in nitrate treatment were 14 cm and 14 cm during the first and second weeks and longest among treatments. Those in ammonium treatment were 4 cm and 1 cm during the first and second weeks, and most strongly inhibited among treatments. The marked lateral root length in the urea and Gln treatments was slightly lower than control treatment either in the first and second weeks.

**FIGURE 11:**
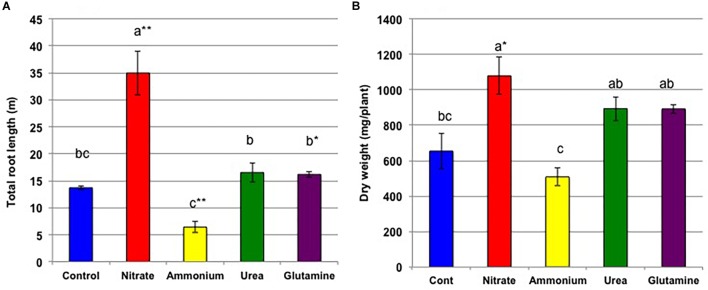
Total root length **(A)** and the dry weight of roots **(B)** on 34 DAP after 2 weeks of the treatments with various forms of N compounds (Experiment 4). Averages and standard errors are shown (*n* = 3). Different letters above the column indicate significant differences among each treatment at <0.05 by Tukey’s test.

Figure 11 shows the total root length analyzed by WinRHIZO (Figure 11A) and the dry weight of the roots (Figure 11B). Both figures show the significant increase in the total root length and root dry weight in nitrate treatment compared with control treatment. The ratio of the total root length (nitrate/control) is x2.4-fold and much higher than the ratio of the dry weight (x1.6) indicate that fine roots growth was promoted by nitrate treatment. On the other hand, the total root length and root dry weight were lower in the ammonium treatment compared with the control treatment. The total root length and dry weight in urea and Gln treatments were slightly higher than the control roots. Supplementary Figure S1 shows the photos of the root system with various N treatments after 1 week treatment on 27 DAP. The nitrate promoted the fine lateral root growth, while ammonium depressed new root growth compared with the control plant. The roots with urea and Gln treatments show the increased root growth between control and nitrate treatments.

After 2 weeks N treatments on 34 DAP, the nodule dry weight was highest in control, followed by urea, Gln, nitrate and ammonium treatment (Supplementary Figure S4A). On the other hand, the repression of three selected nodule growth were the highest in ammonium and nitrate, followed by urea and Gln (Supplementary Figure S4B). Nodule numbers are around 40 to 65, and not significantly different among treatments.

Two weeks of the long-term nitrogen treatments affected the whole plant growth (Supplementary Figure S5). After 2 weeks N treatments on 34 DAP, the average total plant dry weight was 2,008 mg (control), 3,114 mg (nitrate), 1,755 mg (ammonium), 2,569 mg (urea), and 2,612 mg (Gln). Nitrate treatment showed the highest growth promotion of leaves, stems, petioles, and buds but not for nodules. Urea and Gln treatments moderately increased the plant growth compared with the control, but ammonium repressed the growth of each organ. Supplementary Figure S6 shows the photos of shoot organs among treatments, and Supplementary Figure S7 shows the total leaf area analyzed by WinRHIZO. These figures clearly show that nitrate promoted the leaf growth as well as root growth. Urea and Gln treatment showed moderate promotion of leaf growth compared with the control plants. The ammonium treatment repressed the leaf growth as well as the root growth. Supplementary Figure S8 shows the SPAD value of the second, third, and fourth leaves with various N treatments. The SPAD values with Gln were the highest followed by those with urea, nitrate, control, and ammonium. The 2 weeks nitrogen treatment markedly affected the bud development, and the average number of the lateral buds was 1 in control, 3.7 in nitrate, 0 in ammonium, 2 in urea, and 5 in Gln (Supplementary Figure S6).

## Discussion

### Effects of Various Nitrogen Compounds on Nodule Growth and Acetylene Reduction Activity

From the daily changes of nodule volume from 12 to 17 DAP with following supplementation (1 mM-N nitrate, ammonium, urea, and Gln), and the continuation of cultivation with the N-free culture solution from 17 to 24 DAP (Figure 3), quick and reversible inhibitory effects on nodule growth were evident for ammonium, urea, and Gln supplementation. The inhibitory effects of ammonium were as high as that for nitrate during the period from 12 to 17 DAP, whereas the inhibitory effects for urea and Gln were less compared to that for ammonium and nitrate treatments. After a change of the culture solution to an N-free solution, recovery of nodule growth was observed from the next day in the case of urea, Gln, and ammonium in contrast to that for nitrate treatment where there was a 2 days time-lag. In this experiment a 1 mM-N supplementation was used, but the inhibitory effect of nitrate on nodule growth was similar to a previous experiment using 5 mM nitrate (Fujikake et al., 2002, 2003).

The effect of the nitrogen compounds on the nodule dry weight (Figure 1A) and the ARA per a single plant (Figure 2A) at 17 DAP after the N treatments resulted in significant repression in growth compared to that for the control. The specific ARAs were not inhibited compared with the control after 5 days of supplementation with the various forms of N (Figure 2B) indicate that decline in total ARA is mainly due to reduced nodule dry weight. After 7 days of N-free cultivation (17–24 DAP), the nodule dry weights of the plants for ammonium, urea, and Gln supplementation recovered to the same levels as that for the control nodules (Figure 4A), although that for the nitrate-treated plants tended to be low. The ARAs per single plant at 24 DAP after 7 days of N-free cultivation did recover (Figure 5A), although nitrate supplementation resulted in the lowest ARA. It was surprising that the Gln treatment gave the highest ARA at 24 DAP, about two times higher than that of the control plants. The specific ARA per nodule dry weight for Gln supplementation was also significantly higher for the other treatments including that for the control nodules (Figure 5B). The reason for the promotion of specific ARAs by Gln at the physiological level is unknown.

### Absorption and Transport of Various Chemical Forms of N in Soybean Plants

The total N derived from the ^15^N-labeled sources were 9.75 mg (nitrate), 8.71 mg (ammonium), 7.04 mg (urea), and 9.67 mg (Gln), respectively. The percentage distribution of ^15^N in the shoots (leaves + stems) was high for nitrate (67%) and ammonium (65%) treatments compared with urea (57%) and Gln (49%) (Supplementary Table S2). These results indicated that all N compounds including urea and Gln were actively absorbed in soybean roots and transported to the shoots in 3 days, although translocation of the absorbed N to the shoots was relatively slower for urea and Gln compared to that for nitrate and ammonium. In addition, the percentage distribution of ^15^N in the nodules for nitrate (1.5%), ammonium (2.9%), Gln (3.0%) and urea (1.9%) treatments was relatively low among the tissues and appeared not to be directly related to the inhibitory effect on nodule growth and the ARA.

In an independent study (Ohyama, 1983), the transport of fixed ^15^N_2_ in the nodules and absorbed ^15^NO_3_^-^ in the roots were compared at the pod filling stage. About 36% of the fixed N was found to remain in the nodules, and the rest was distributed in the roots (9%), stems (17%), leaves (18%), pods (10%) and seeds (10%) after just 10 h of ^15^N_2_ treatment. In contrast, 36% of the absorbed ^15^NO_3_^-^ remained in the roots, and the rest was contained in the nodules (0.4%), stems (17%), leaves (36%), pods (5%) and seeds (5%) after 10 h of supplementation by ^15^NO_3_^-^. These results suggested that the transport rates from source organs, either the nodules or the roots, were similar, but fixed-N was more rapidly transported to the pods and seeds; also the absorbed nitrate was highly distributed in the leaves. The nodulated soybean plants were treated with a culture solution containing ^15^NO_3_^-^, ^15^NO_2_^-^, or ^15^NH_4_^+^, and the absorption and transport of N were investigated (Ohyama et al., 1989b). After 24 h of ^15^N-supply, the amount and distribution of ^15^N among the roots, nodules, stems, and leaves were very similar between ^15^NO_3_^-^ and ^15^NH_4_^+^, although the absorption and transport to the shoot from ^15^NO_2_^-^ was much lower than that from ^15^NO_3_^-^ and ^15^NH_4_^+^ and most of the ^15^N from the ^15^NO_2_^-^ remained in the roots.

In the present experiment (Figure 7), the absorption and distribution of ^15^N among the tissues were relatively similar for ^15^NO_3_^-^ and ^15^NH_4_^+^. Absorption of urea tended to be lower than for the other compounds but urea and Gln were transported to the stems and leaves. The principal N compounds transported via the xylem from the nodules are ureides (allantoin and allantoic acid), which account for 80–90% of the total N in the xylem sap collected from nodulated soybean cultivated under N-free conditions. However, the xylem sap collected from non-nodulated soybean contains mainly amino acids and nitrate with a small portion of ureides (10–20%) (Ohyama et al., 2012). Asn is the principal amino acid in xylem sap collected from field grown soybean plants (Ohtake et al., 1995; Sato et al., 1998). Vitor et al. (2018) reported that Asp was the prominent amino acid component of phloem sap, followed by Glu, Asn, and Ser either in the nodulated or non-nodulated soybean cultured with nitrate or ammonium. The absorbed N supplied by ^15^NO_3_^-^ was very rapidly transported through the xylem (Ohyama et al., 1989a), and the absorption and transport of ^13^NO_3_^-^ were not affected by nodulation in the roots observed by a positron emitting tracer imaging system (Sato et al., 1999).

### Nitrogen Metabolism

The total free amino acid concentration increased in all plant tissues for the various nitrogen treatments (Figure 9). The most prominent increases in tissue concentrations, irrespective of N-forms, were Asn and Asp. In Gln treatment, the free Asn concentration was higher than that of Gln, suggesting that the absorbed Gln in the roots was readily converted to Asn and transported in the forms of Asn and Asp. Most of the ammonium in the culture solution may have been assimilated in the roots by the GS/GOGAT (glutamine synthetase/glutamate synthase) pathway to Gln, then Asn was formed from Gln and Asp by the enzyme AS. A part of nitrate absorbed in the roots is initially reduced by NR and NiR to ammonium and then assimilated into Gln via the GS/GOGAT pathway. The remaining nitrate may have been translocated to the leaves and reduced and assimilated there. Absorbed urea may have been hydrolyzed to ammonium and carbon dioxide by urease, with ammonium then being assimilated via the GS/GOGAT pathway. The absorbed Gln in the roots may have been assimilated into Asn by AS.

### Absorption and Metabolism of Urea

Urea may be absorbed either directly from roots, or after urea degradation by soil microbes. It has been recognized that urea is a plant metabolite derived either from root uptake or from catabolism of Arg by arginase (Witte, 2011). In soybean plants, it is reported that urea may be produced during ureide degradation (Tod and Polacco, 2004). The present experiments use hydroponics without soil micro-organisms, therefore, urea might be directly absorbed by the roots, although plants have not been aseptically cultivated. Plants possess a high affinity secondary active urea transporters (DUR3) and passive transporters (MIPs). Urease is a unique nickel enzyme and it hydrolyzes urea to ammonia and carbamate, and the carbamate rapidly decays to ammonia and carbon dioxide non-enzymatically. Soybean has two ureases, a highly expressed embryo-specific urease encoded by the *Eu1* gene (Meyer-Bothling et al., 1987; Witte, 2011), and a ubiquitous urease found in all tissues encoded by *Eu4* (Torisky et al., 1994; Witte, 2011).

### Absorption and Metabolism of Glutamine

Amino acids can be absorbed and utilized in *Arabidopsis*, and the supply of L-Gln and L-Asn promotes plant growth (Forsum et al., 2008). Hirner et al. (2006) reported that *Arabidopsis* LHT1 is a high-affinity broad specificity plasma membrane-bound transporter for cellular amino acid uptake in both roots and leaves. The H^+^-independent Gln transport in the root tips of *Arabidopsis* has also been reported (Yang et al., 2010).

### Involvement of Carbon Supplementation in the Inhibition of Nodule Growth and Nitrogen Fixation Activity by Nitrogen Compounds

The percentage distribution of ^13^C in nodules was highest in control treatment (11.5%), which was higher than that of urea (5.8%), Gln (2.6%), ammonium (2.3%), and nitrate (2.3%) (Supplementary Table S3). The decrease in photosynthetic carbon supply seems to cause the repression of nodule growth (Figure 3) and a decrease in the ARA (Figure 2), irrespective of N forms. In previous experiments, where soybean shoots were exposed to ^14^C-labeled CO_2_ for 2h, the addition of 5 mM of nitrate to the culture solution stimulated the translocation of labeled-C to the roots from 5.2 to 9.1% of total photoassimilate and decreased the partitioning of C to the nodules from 9.1 to 4.3% just after ^14^CO_2_ exposure (Fujikake et al., 2003). These findings indicated that a decreased supply of photoassimilate to nodules might be involved in the quick but reversible nitrate inhibition of nodule growth and its nitrogen fixation activity. In addition, the inhibitory effect of nitrate was alleviated by the addition of sucrose to the medium. These results indicated that the decrease in photoassimilate supply to nodules may be involved in the quick and reversible nitrate inhibition of soybean growth. The decrease in photoassimilate partitioning to the nodule may be caused by the increased photoassimilate consumption in the roots to absorb and assimilate N sources for providing energy and carbon skeletons of amino acids. In addition, N sources might affect root growth rate and the architecture (Kiba and Krapp, 2016).

In the continuous long-term N treatments for 2 weeks from 20 to 34 DAP, we observe the difference of root architecture by N sources, especially nitrate significantly promoted the lateral root growth, with moderate promotion by urea and Gln but repressed the root growth by ammonium (Figure 10, 11). The promotion of lateral root growth by nitrate, urea, and Gln was in accordance with increase in leaf growth (Supplementary Figures S3, S4) and increase in chlorophyll contents (Supplementary Figure S6). The depressive effects by ammonium on root and leaf was not observed in Experiment 1 (Figure 1) and Experiment 2 (Figure 4). The repression by ammonium in Experiment 4 may be long-term ammonium supply gave ammonium toxicity on the roots, or the decrease in the pH in solution might affect the roots. In Experiment 1 and 2 the culture solution was changed every day, but in Experiment 4 the culture solution was changed every 2 days.

Very recently, transcriptome and metabolome analyses have revealed that nitrate supply promoted nitrogen and carbon metabolism in soybean roots, but tends to repress nitrogen and carbon metabolism in the nodules (Ishikawa et al., 2018). A microarray-based transcriptome analysis was carried out on soybean roots and nodules after 24 h of 5 mM nitrate treatment. Nitrate treatment enhanced substantially the gene expression of nitrate transport and the nitrogen assimilation pathways in the roots, but much less so in the case of nodules. Gene expression related to glycolysis and the TCA cycle in the roots were also increased but those in the nodules were repressed after 24 h of nitrate treatment. Metabolome analysis of roots and nodules were in accordance with transcriptome analysis, and both analyses support the hypothesis that nitrate supply promotes carbon use in the roots and as a result decreases carbon transport to the nodules.

### Models of Absorption, Metabolism, and Transport of Various N Forms in a Nodulated Soybean Plant

**FIGURE 12:**
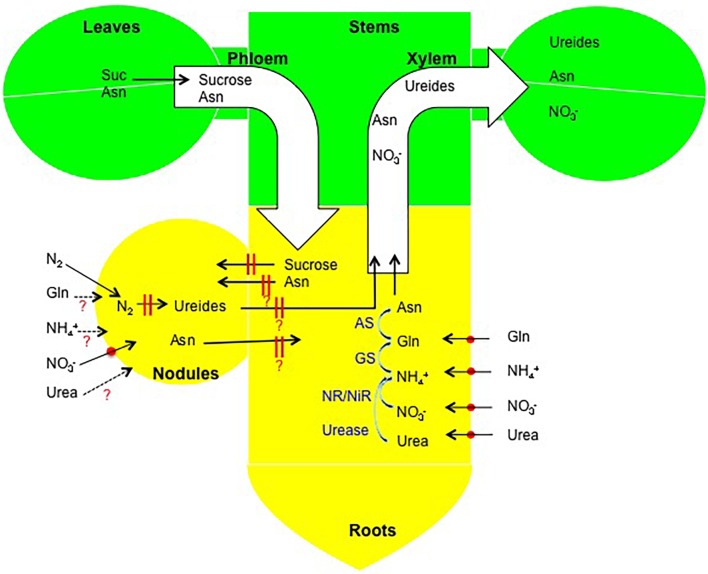
Outline of the absorption, metabolism, and transport of various forms of N in soybean plants.

Figure 12 gives an outline of the flow of fixed-N in the nodules and the N-compounds supplied to the culture solution. Ammonium (NH_4_^+^), nitrate (NO_3_^-^), urea, and Gln are absorbed by ammonium transporter, nitrate transporter, urea transporter, and amino acid transporter in the cell membrane of the epidermal or cortical cells of the roots. A high accumulation of NH_4_^+^ is known to be toxic to plants, so most of NH_4_^+^ absorbed in the roots should be assimilated rapidly to Gln by GS. The Gln is converted to Asn and Asp in the roots, then transported to the shoots via the xylem. The absorbed Gln from solution can be assimilated in the same way as NH_4_^+^. A portion of NO_3_^-^ absorbed in the roots is reduced by NR to NO_2_^-^ and the NO_2_^-^ is reduced to NH_4_^+^ by NiR in the roots, then assimilated the same as for NH_4_^+^. The other part of NO_3_^-^ is transported to the shoot via the xylem and reduced and assimilated in the leaves. The absorbed urea in the roots may be hydrolyzed to NH_4_^+^ in the roots, but some urea may be transported to the shoots and metabolized in the stems and leaves. It was shown that NO_3_^-^ is absorbed from the nodule surface (Mizukoshi et al., 1995), possibly by the NO_3_^-^ transporter, because nitrate transporter genes were promoted after 24 h of NO_3_^-^ treatment (Ishikawa et al., 2018). It is not known whether NH_4_^+^, urea, and Gln are absorbed from nodule surface.

The decrease in nitrogen fixation activity may be caused by reduced photoassimilate supply, the same as for nodule growth inhibition. However, another possibility, Asn accumulation in nodules by nitrate in this study (Figure 9A) and the results by metabolome (Ishikawa et al., 2018) may be related to the a decrease in nitrogen fixation activity possibly through decreased oxygen permeability of the nodule cortex (Serraj et al., 1999) or other reasons. Further research is required to obtain a comprehensive understanding of the complex mechanisms and regulation by nitrogen and carbon for nodule growth and nitrogen fixation.

Concerning to the lateral root growth, Lavenus et al. (2013) reviewed that phytohormone auxin act as a common integrator to many endogenous and environmental signals regulating lateral root formation in model plant *Arabidopsis thaliana*. The changes in root and shoot growth by various N sources may be regulated by phytohormones such as auxin, cytokinin, ethylene, abscisic acid, gibellerines, brassinolide, etc., but the evidence had been not obtained for the relationship between N compounds and hormones in soybean related to lateral root growth and nodule growth as well.

## Conclusion

A rapid and reversible repression of nodule growth and nitrogen fixation activity was observed by ammonium, urea, or Gln supply to the culture solution similar to that for nitrate, although the effect was milder for urea and Gln supply compared for nitrate and ammonium treatments. Nitrogen from ammonium, urea, and Gln was actively absorbed by the soybean roots and more than half of the absorbed N was transported to the shoot during the 3 days of application period. The application of the different chemical forms of N increased the free amino acids contents in each tissue, and Asn and Asp were the dominant amino acids. The distribution of labeled-C and not labeled N in the nodules correlated with the repression of nodule growth, nitrogen fixation activity, and nodule growth. It was found that the Gln treatment followed by N-free cultivation gave the highest nitrogen fixation activity, about two times higher than that of the control plants. The long-term supply of various N forms for 2 weeks, nitrate significantly increased plant growth, especially lateral root growth and leaf growth. The long-term supply of urea and Gln also promoted the lateral roots and leaf growth, but ammonium suppressed plant growth including root growth and leaf growth.

## Author Contributions

TO conceived the research, designed the experiments, analyzed the data, and wrote the manuscript. NY and ST carried out all the experiments and analysis. NO, KS, TS, KH, and AS gave valuable suggestions during the experiments and writing of the manuscript.

## Conflict of Interest Statement

The authors declare that the research was conducted in the absence of any commercial or financial relationships that could be construed as a potential conflict of interest. The handling editor and reviewer BF declared their involvement as co-editors in the Research Topic, and confirm the absence of any other collaboration.

## Supplementary Material

Figure S1 ^13^CO_2_ injection into a plastic bag covering the plant shoot (Experiment 3) **(A)**. CO_2_ concentration was monitored by infrared CO_2_ analyzer **(B)**.

Figure S2 Nodule number per single plant at 17 DAP supplied with various nitrogen compounds from 12 to 17 DAP (Experiment 1). Averages and standard errors are shown (*n* = 4). Different letters above the column indicate significant differences at <0.05 by Tukey’s test.

Figure S3 Photos of root systems with various N treatments for 1 week (Experiment 4). White square is 3 cm × 3 cm.

Figure S4 Dry weight of nodules **(A)** after 2 weeks of N treatments, and the increase in the volume of three selected nodules in the first and the second week **(B)** (Experiment 4). Averages and standard errors are shown (*n* = 3). Different letters above the column indicate significant differences at <0.05 by Tukey’s test.

Figure S5 Dry weight of each organs after 2 weeks of various N treatments (Experiment 4).

Figure S6 Photos of shoot organs with various N treatments for 2 week. White square is 3 cm × 3 cm (Experiment 4).

Figure S7 Total leaf area per a plant after 2 weeks of various N treatments (Experiment 4). Averages and standard errors are shown (*n* = 3). Different letters above the column indicate significant differences at <0.05 by Tukey’s test. ^∗^Indicate significantly differences between control at <0.05 by Student’s *T*-test.

Figure S8 SPAD value of the second, third, and fourth trifoliolate leaves after 2 weeks of various N treatments (Experiment 4). Averages and standard errors are shown (*n* = 3). Different letters above the column indicate significant differences at <0.05 by Tukey’s test.

Table S1 Dry weight of each tissue of soybean plants (Experiment 3).

Table S2 Percentage distribution of ^15^N labeled source among each tissue of soybean plants (Experiment 3).

Table S3 Percentage distribution of ^13^C among each tissue of soybean plants (Experiment 3).

Table S4 Nitrogen concentration of free amino acids in each tissue of soybean plants treated with N compounds for 3 days (Experiment 3).

Table S5 Relative concentration of free amino acids in each tissue of soybean plants treated with N compounds relative to control plants (Experiment 3).

## Abbreviations

Ala: alanine
ARA: acetylene reduction activity
Arg: arginine
AS: asparagine synthetase
Asn: asparagine
Asp: aspartic acid
Cys: cysteine
DAP: days after planting
GABA: 4-aminobutanoic acid
Gln: glutamine
Glu: glutamic acid
Gly: glycine
GOGAT: glutamine 2-oxoglutarate aminotransferase (glutamate synthase)
GS: glutamine synthetase
His: histidine
Ile: isoleucine
Leu: leucine
Lys: lysine
Met: methionine
NR: nitrate reductase
NiR: nitrite reductase
Phe: phenylalanine
Pro: proline
Ser: serine
Trp: tryptophan
Tyr: tyrosine
Val: valine
