# Supplementary figures and images for: Effects of Different Chemical Forms of Nitrogen on the Quick and Reversible Inhibition of Soybean Nodule Growth and Nitrogen Fixation Activity
Source: Front Plant Sci. 2019 Feb 19;10:131. doi: 10.3389/fpls.2019.00131 (PMC6389793; doi:10.3389/fpls.2019.00131)

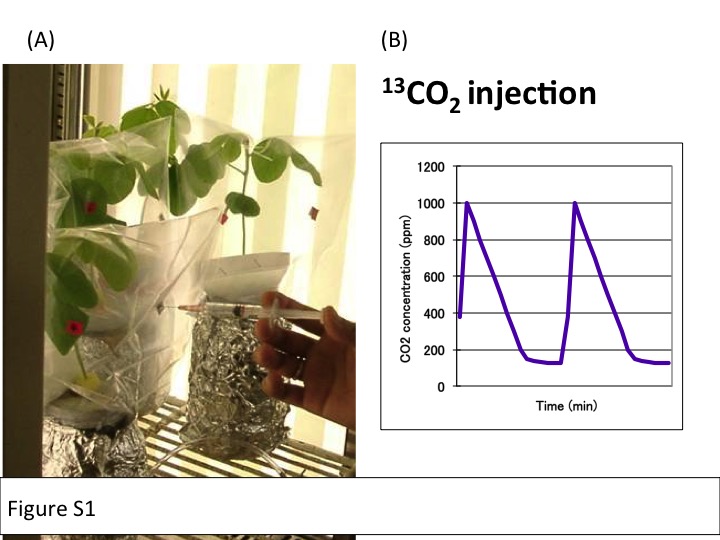

Supplement: Figure S1 — 13CO2 injection into a plastic bag covering the plant shoot (Experiment 3) (A). CO2 concentration was monitored by infrared CO2 analyzer (B). [file Image_1.JPEG]

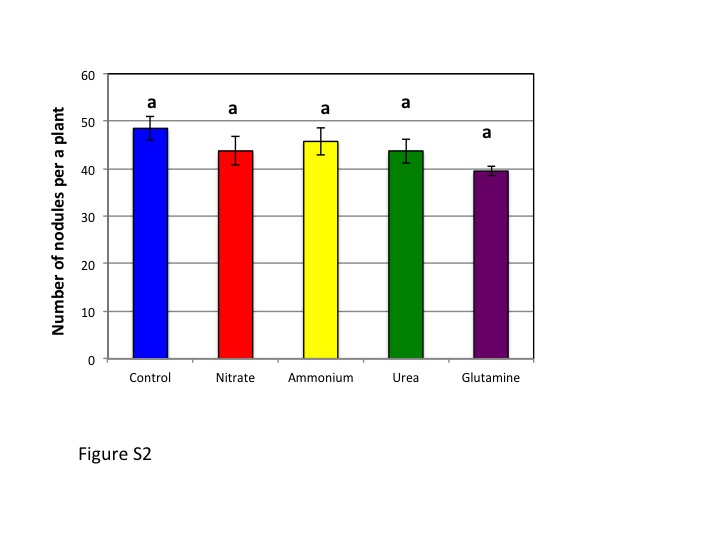

Supplement: Figure S2 — Nodule number per single plant at 17 DAP supplied with various nitrogen compounds from 12 to 17 DAP (Experiment 1). Averages and standard errors are shown (n = 4). Different letters above the column indicate significant differences at <0.05 by Tukey’s test. [file Image_2.JPEG]

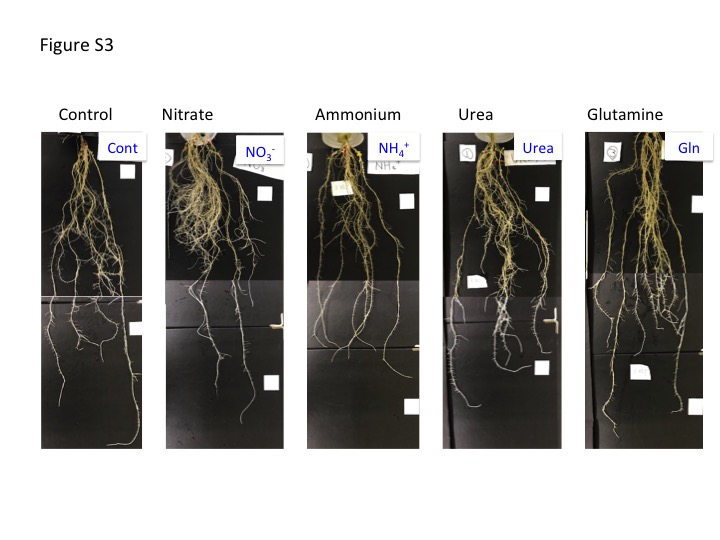

Supplement: Figure S3 — Photos of root systems with various N treatments for 1 week (Experiment 4). White square is 3 cm × 3 cm. [file Image_3.JPEG]

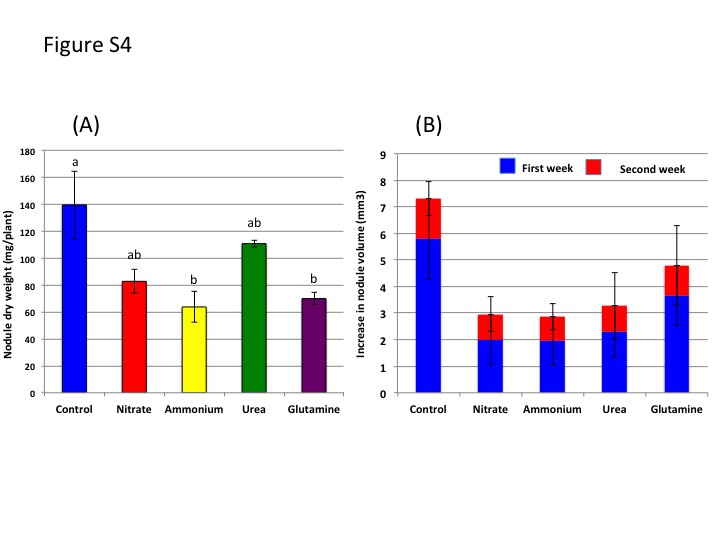

Supplement: Figure S4 — Dry weight of nodules (A) after 2 weeks of N treatments, and the increase in the volume of three selected nodules in the first and the second week (B) (Experiment 4). Averages and standard errors are shown (n = 3). Different letters above the column indicate significant differences at <0.05 by Tukey’s test. [file Image_4.JPEG]

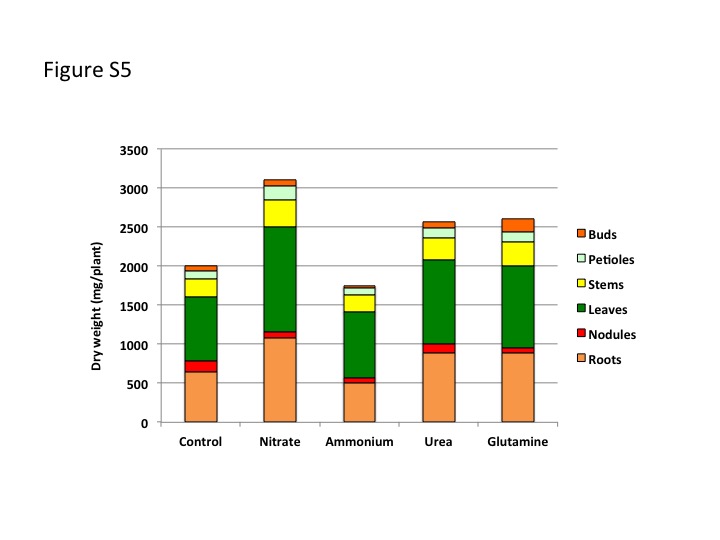

Supplement: Figure S5 — Dry weight of each organs after 2 weeks of various N treatments (Experiment 4). [file Image_5.JPEG]

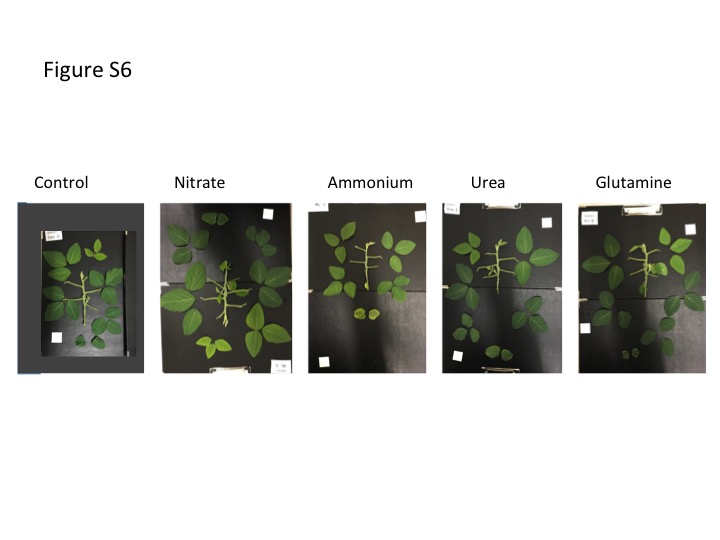

Supplement: Figure S6 — Photos of shoot organs with various N treatments for 2 week. White square is 3 cm × 3 cm (Experiment 4). [file Image_6.JPEG]

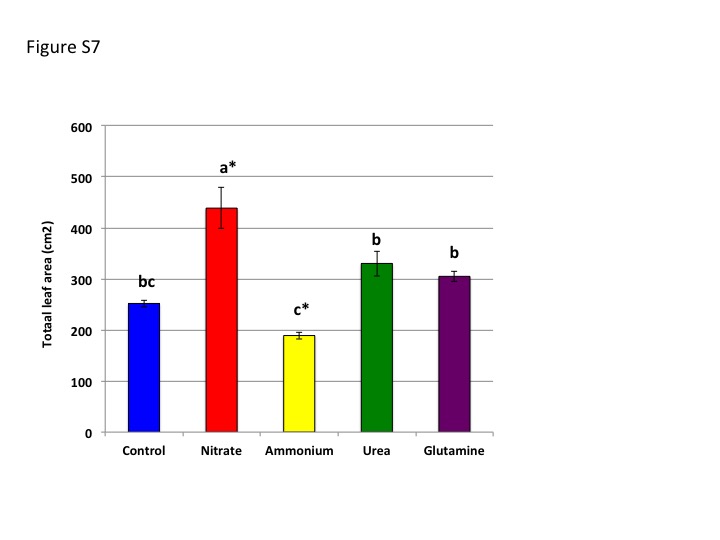

Supplement: Figure S7 — Total leaf area per a plant after 2 weeks of various N treatments (Experiment 4). Averages and standard errors are shown (n = 3). Different letters above the column indicate significant differences at <0.05 by Tukey’s test. ∗Indicate significantly differences between control at <0.05 by Student’s T-test. [file Image_7.JPEG]

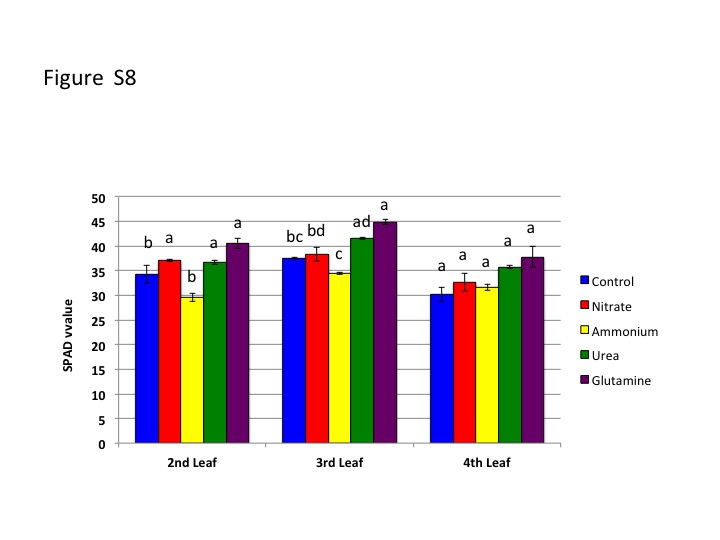

Supplement: Figure S8 — SPAD value of the second, third, and fourth trifoliolate leaves after 2 weeks of various N treatments (Experiment 4). Averages and standard errors are shown (n = 3). Different letters above the column indicate significant differences at <0.05 by Tukey’s test. [file Image_8.JPEG]
